# Screening and diagnosis of sarcopenia in rheumatic and musculoskeletal diseases: findings from a cross-sectional study

**DOI:** 10.1007/s00296-025-05821-7

**Published:** 2025-02-28

**Authors:** Eleni C. Pardali, Katerina-Maria Kontouli, Arriana Gkouvi, Irene A. Tsakmaki, Eleni Patrikiou, Maria Karapli, Christos Liaskos, Nektarios Marios Liapis, Vasiliki Syrmou, Ioannis Alexiou, Theodora Simopoulou, Sousana K. Papadopoulou, Christina G. Katsiari, Efterpi Zafiriou, Dimitrios G. Goulis, Dimitrios P. Bogdanos, Maria G. Grammatikopoulou

**Affiliations:** 1 Unit of Immunonutrition and Clinical Nutrition, Department of Rheumatology and Clinical Immunology, Faculty of Medicine, School of Health Sciences, University of Thessaly, Biopolis campus, Biopolis, Larissa Greece; 2 Department of Primary Education, School of Education, University of Ioannina, Ioannina, Greece; 3 Department of Nutritional Sciences and Dietetics, School of Health Sciences, International Hellenic University, Alexander Campus, Thessaloniki, Greece; 4 Department of Dermatology, Faculty of Medicine, School of Health Sciences, University General Hospital of Larissa, University of Thessaly, Biopolis Campus, Larissa, Greece; 5 Unit of Reproductive Endocrinology, 1st Department of Obstetrics and Gynecology, Medical School, Aristotle University of Thessaloniki, Papageorgiou General Hospital, Thessaloniki, Greece

**Keywords:** Myopenia, Systemic sclerosis, Fat-free mass, Handgrip strength, Systemic lupus erythematosus, Rheumatoid arthritis, Body composition, Inflammation

## Abstract

Sarcopenia is characterized by loss of muscle mass and reduced muscle function, presenting various adverse events, especially when inflammation is present. The present study aimed to determine the prevalence of sarcopenia and sarcopenic obesity among patients with rheumatic and musculoskeletal diseases (RMDs) and identify the risk for sarcopenia using two screening tools the Strength, assistance with walking, rising from a chair, climbing stairs, and falls (SARC-F) and the Mini Sarcopenia Risk Assessment (MSRA). In this single-center cross-sectional study, 220 consecutive patients visiting the Department of Rheumatology and Clinical Immunology at the University General Hospital of Larissa were interviewed. The EWGSOP criteria were used for the diagnosis of sarcopenia, while sensitivity, specificity, positive predictive values, number needed to screen, and positive and negative likelihood ratios were used to validate the diagnostic validity of the SARC-F and the MSRA. Univariate and multivariate logistic regression analyses were also applied to model the relationship between sarcopenia and other variables. In the total sample, 15.9% of patients were diagnosed with sarcopenia and one patient with sarcopenic obesity. The SARC-F (sensitivity 22.2%, specificity 75.6%), the 5-item (sensitivity 88.9%, specificity 18.9%), and the 7-item MSRA (sensitivity 91.7%, specificity 9.2%) presented poor clinical performance when used for screening alone. Univariate logistic regression analyses showed that underweight status, systemic sclerosis and appetite loss are strong contributors to sarcopenia diagnosis. Sarcopenia is prevalent among RMDs, and screening is essential within RMD clinics. None of the screening tools (SARC-F and MSRA) can stand alone in assessing sarcopenia in patients with RMDs. More research is required to understand sarcopenia in RMDs and validate the wide-using screening tools.

## Introduction

Sarcopenia is a multifactorial skeletal muscle disorder characterized by a progressive decline of muscle function and loss of muscle mass [1]. It is associated with loss of autonomy, increased fall risk, and frailty [1]. The European Working Group on Sarcopenia in Older People (EWGSOP) criteria [2, 3] for sarcopenia are widely used for its diagnosis; however, a universally agreed-upon definition has yet to be established. The EWGSOP recommends the coinciding presence of reduced muscle mass and low muscle function, demonstrated by limited strength and impaired performance [2]. Along with the criteria, the Strength, Assistance with walking, rising from a chair, Climbing stairs and Falls (SARC-F) screening tool [4] was introduced to identify individuals at risk of developing sarcopenia. On the other hand, the Mini Sarcopenia Risk Assessment (MSRA) [5] questionnaire serves as another screening tool for this purpose.

Rheumatic and musculoskeletal diseases (RMDs) consist of several distinct chronic disorders characterized by inflammation driven by overactive innate immune responses [6]. Their heterogeneous symptomatology involves pain, decreased physical activity, and poor quality of life [6]. Persistent inflammation and limited physical function are significant contributors to sarcopenia [2, 7], placing individuals with RMDs at a heightened risk for becoming sarcopenic. In addition, low body mass index (BMI) and the use of medication have also been characterized as contributors to sarcopenia [7]. Several studies have researched the prevalence of sarcopenia in RMDs using different methods of assessment, reporting varied outcomes such as malnutrition [8, 9], osteoporosis [9], disease severity [7, 9, 10], and frailty [11], among others. The diagnosis of sarcopenia in this population differs depending on the criteria used, while the overall risk of sarcopenia has not been widely assessed. Although the SARC-F tool has been applied in some studies [7, 12–16], the MSRA tool has yet to be evaluated in this population.

### Objectives

The present cross-sectional study aimed to determine the prevalence of sarcopenia and sarcopenic obesity in individuals diagnosed with RMDs and evaluate the validity of two screening tools for sarcopenia assessment within this population.

## Methods

### Study design and sample

A total of 220 consecutive inpatients and outpatients visiting the Department of Rheumatology and Clinical Immunology at the University General Hospital of Larissa were recruited in this cross-sectional study, from July 2023 to October 2024. Sample size was not calculated, as the data pertain to a specific hospital in a defined region, and no exclusion criteria were applied, except for excluding those who declined to participate. Inclusion criteria were (i) adult patients diagnosed with one or more rheumatic diseases and (ii) ability to communicate in the Greek language. Patients were interviewed as inpatients or outpatients during intravenous (IV) immune therapy sessions. The characteristics of the patients are presented in Table 1. All patients provided informed consent before participation. For practical purposes, rheumatoid arthritis (RA) and juvenile arthritis (JA) were grouped under the term “arthritis”. At the same time, Henoch-Schönlein purpura, Churg-Strauss syndrome, polyangiitis, Wegener’s granulomatosis, Buerger’s disease, Cogan syndrome, and giant cell arteritis (GCA) are classified as vasculitides. Dermatomyositis (DM), along with myositis, were categorized as myositides. Other diagnoses of the sample included systemic lupus erythematosus (SLE), axial spondylarthritis (SpA), systemic sclerosis (SSc), psoriatic arthritis (PsA) and many more (Table 1). Blood parameters of each patient were recorded from the patient’s hospital files.

**Table 1:** Characteristics of the study population (*N* = 220)

Variables	Categories and units	Values
Sex	Females/Males (*n*, %)	154 (70)/ 66 (30)
Age (age)	Females/Males (*n*, %)	59.7 ± 14.1/59.5 ± 17.8^∝^
Education	Primary/Middle School/Tertiary/Postgraduate (*n*, %)	97 (44.1)/ 73 (33.2)/ 46 (20.9)/ 3 (1.4)
Employment	Full-time/Part-time/Unemployed /Retired/Ill-health retirement (*n*, %)	56 (25.5)/12 (5.5)/ 44 (20)/ 89 (40.5)/ 18 (8.2)
Ethnicity	Caucasian/Roma/Latino/African American (*n*, %)	213 (96.8)/5 (2.3)/ 1 (0.5)/ 1 (0.5)
Current smokers	(*n*, %)	47 (21.4)
Central obesity	(*n*, %)	129 (58.6)
BMI (kg/m^2^)		27.9 ± 6.3^∝^
Weight status	Underweight/Normoweight/Overweight-Obese (*n*, %)	11 (5)/ 58 (26.4)/150 (68.2)
Recruitment	Inpatient/Outpatient/IV immunotherapy (*n*, %)	84 (38.2)/91 (41.4)/44 (20)
Dysphagia	(*n*, %)	64 (29.1)
Appetite loss	(*n*, %)	25 (11.4)
CRP (mg/dL)		5.1 ± 29.6^∝^
ESR (mm/h)		41.9 ± 37.1^∝^
On Corticosteroids	(*n*, %)	81 (36.8)
RMDs	Arthritis (RA, JA) (*n*, %)	79 (35.9)
	Vasculitides (Henoch-Schönlein, Churg-Strauss, polyangiitis, Wegener, Behcet, Buerger’s, Cogan, GCA, Takayasu) (*n*, %)	32 (14.5)
	SLE (*n*, %)	28 (12.7)
	PsA (*n*, %)	21 (9.6)
	SpA (*n*, %)	17 (7.7)
	SSc (*n*, %)	17 (7.7)
	Myositides (*n*, %)	8 (3.6)
	Yet Undiagnosed (*n*, %)	12 (5.5)
	Sjogren’s disease (*n*, %)	7 (3.2)
	Sarcoidosis (*n*, %)	3 (1.4)
	APL (*n*, %)	4 (1.8)
	Gout (*n*, %)	4 (1.8)
	FMS (*n*, %)	2 (0.9)
	RPF (*n*, %)	1 (0.5)
	FMF (*n*, %)	1 (0.5)

APL: Antiphospholipid antibody syndrome; AS: ankylosing spondylitis; BMI: Body Mass Index; CRP: C-reactive protein; DM: Dermatomyositis; dL: deciliter; ESR: Erythrocyte Sedimentation Rate; FMF: Familial Mediterranean fever; FMS: Fibromyalgia syndrome; GCA: Giant cell arteritis; hr: hour; IV: Intravenous; JA: Juvenile arthritis; mg: milligrams; ml: millimeters; PsA: psoriatic arthritis; RA: rheumatoid arthritis; RMDs: Rheumatic musculoskeletal disease; RPF: retroperitoneal fibrosis; SD: standard deviation; SLE: Systemic Lupus Erythematosus; SpA: spondylarthritis; SSc: Systemic Sclerosis. ^∝^ Mean ± SD

### Data collection

Data and anthropometric measurements were collected from all patients following standardized procedures. Body weight (BW) and height were measured with the use of a digital floor scale (Kern MPE 200 K-1PEM, Kern, Germany) and a stadiometer (Seca 220, Hamburg, Germany) and BMI were calculated for all. Patients were divided into three categories according to their BMI; underweight (< 18.5 kg/m^2^), normoweight (≥ 18.5 kg/m^2^ and < 25 kg/m^2^) and overweight/obesity (≥ 25 kg/m^2^).

Skinfold thickness measurements were recorded at the biceps, triceps, subscapular, and suprailiac sites using a Slimguide caliper (Creative Health, USA) on the right side of the patient’s body. The Jackson and Pollock equations [17, 18] were used for determining body fat (BF, as a percent of body weight), fat-free mass (FFM), fat mass index (FMI), and fat-free mass index (FFMI) [19]. Each body site was measured twice, and the median of the two measurements was used to calculate body composition. Waist circumference was also measured using an anelastic measuring tape.

Handgrip strength (HGS) was assessed using an analog dynamometer (T.Κ.Κ. 5401 Grip-D, Takei, Japan). According to manufacturer guidelines, patients were seated upright with their feet flat on the floor, their elbows fully extended, and their palms facing the body.

### Sarcopenia screening

The SARC-F tool [4, 20] is a component question scoring system screening for sarcopenia, while assessing five distinct components: (i) strength, (ii) assistance with walking, (iii) ability to rise while sitting from a chair, (iv) ability to climb the stairs, and (v) frequency of falls (SARC-F). Each component is rated on a scale from 0 to 2, with responses indicating the difficulty level in performing these activities (none, some, or significant difficulty) and the number of falls experienced during the past year. A score of ≥ 4 out of 10 suggests an elevated risk of sarcopenia [4]. The Greek version of the SARC-F was utilized in this study [20].

The 5- and 7-item MSRA [5] questionnaires were also administered as additional screening tools for sarcopenia, containing 5 and 7 questions, respectively. Both versions assess age, recent weight loss, number of hospitalizations in the past year, meal frequency, and physical performance. The 7-item version additionally evaluates daily dairy product intake and protein sources (e.g., fish, meat, poultry, legumes, and processed meat). Each question has two or three possible answers, assigning a distinct score. In the 7-item MSRA, a score > 30 is considered normal, whereas a score ≤ 30 suggests a risk of sarcopenia. In the 5-item MSRA, a total score ≤ 45 indicates risk of sarcopenia [5]. Herein, the Greek version of the MSRA was applied [21].

### Sarcopenia diagnosis

Sarcopenia was diagnosed based on the criteria established by the EWGSOP [2], using indicators of low muscle strength and low FFMI. Muscle strength was assessed using handgrip strength according to each patient’s BMI [22], and FFMI thresholds were applied as previously outlined (< 18 kg/m^2^ in men and < 15 kg/m^2^ in women) [23].

### Diagnosis of sarcopenic obesity

Low skeletal muscle function and a BMI of ≥ 30 kg/m² were used [24]to assess the prevalence of sarcopenic obesity. Low skeletal muscle mass was defined using the FFMI [25].

### Statistical analyses

Continuous variables were presented as means with their corresponding standard deviations (SD), while categorical variables were represented by their frequencies alongside their percentages. To evaluate the diagnostic performance of the screening tools SARC-F and the 7- and 5-item MSRA, we assessed sensitivity and specificity, positive predictive value (PPV), and the number needed to screen (NNS) against the EWGSOP criteria [2] for sarcopenia. Chi-square tests were used to examine the associations between categorical variables, including the sarcopenia screening results from the SARC-F and MSRA tools and the EWGSOP [2] criteria for sarcopenia. A univariate logistic regression analysis assessed the associations involving sarcopenia evaluation and other covariates such as age, sex, BMI category, smoking status, patient-reported dysphagia and appetite loss, C-reactive protein (CRP), erythrocyte sedimentation rate (ESR), current corticosteroid use, and the presence of RA, SLE, vasculitis, SpA, myositis, SSc, PsA, and any unidentified rheumatic disease.

In cases of large imbalances between groups, we applied the Firth correction to mitigate bias in the univariate logistic regression models. Based on the significant variables identified in the univariate analysis, we performed multivariate logistic regression to adjust for potential confounders. Additionally, we implemented backward elimination to refine the multivariate model, selecting the most relevant variables for the final analysis. Odds ratios (ORs) with their 95% confidence intervals (CIs) were calculated to quantify these associations. A complete-case analysis ensured that the results remained unbiased despite missing data. A *p*-value of less than 0.05 was considered significant for all analyses. Statistical analyses were conducted using R Studio (version 4.4.1) [26].

## Results

### Socio-demographic and clinical characteristics

Most patients were women (70%; *n* = 154) who had retired from work (40.5%; *n* = 89). Nearly all (96.8%) were Caucasians, and 21.4% identified as smokers. Of the 220 patients, 41.4% were interviewed in the outpatient clinic, 38.2% in the inpatient clinic, and 20% during IV immunotherapy. Regarding their weight status, 68.2% were overweight or obese, and 5% were underweight, while 58.6% had central obesity. Based on self-reports, 29.2% had dysphagia, 11.4% had appetite loss, and 36.8% were on current corticosteroids. The mean (± SD) CRP (mg/dL) and ESR (mm/h) levels were 5.1 (± 29.6) and 41.9 (± 37.1), respectively. In terms of disease distribution, 35.9% were diagnosed with RA, 14.5% with vasculitides, and 12.7% with SLE.

### Prevalence of sarcopenia, sarcopenic obesity and diagnosing performance of the screening tools

The prevalence of sarcopenia (Table 2) using the EWGSOP criteria was 15.9%. Οf the patients, 66.5% (*n* = 147) fell short of the threshold values for HGS, while 21.7% (*n* = 48) were below the cut-off values for FFMI. Sarcopenia-positive screening using the SARC-F reached 23.9% (*p* = 0.787), indicating no association in detecting the condition. The 7- and 5-item MSRA had a prevalence of 90.9% (*p* = 0.870) and 82.3% (*p* = 0.261) respectively, again denoting a lack of association. The chi-squared analyses are presented in Table 3.

**Table 2:** Prevalence of sarcopenia risk and diagnosis using different methods (Ν = 220)

	Variables	Prevalence *n* (%)	*p* value
Diagnosis of sarcopenia (EWGSOP criteria)		36 (15.9)	
Risk of sarcopenia	SARC-F	53 (23.9)	0.787^¥^
	5-item MSRA	182 (82.3)	0.261^¥^
	7-item MSRA	201 (90.9)	0.870^¥^

EWGSOP: European Working Group on Sarcopenia in Older People [2, 3]; MSRA: Mini Sarcopenia Risk Assessment [5]; SARC-F: Strength, Assistance with walking, Rise from a chair, Climb stairs and Falls [4]; ^¥^Chi-squared test comparing each variable with sarcopenia diagnosis based on EWGSOP criteria [2]

**Table 3:** Diagnostic performance metrics for SARC-F and MSRA screening tools (Ν = 220)

	Specificity^§^	Sensitivity^§^	PPV^§^	NNS	LR+	LR-
SARC-F score ≥ 4	75.6	22.2	15.1	83	0.9	1.03
7-item MSRA score ≤ 30	9.2	91.7	16.4	-779	1	0.98
5-item MSRA score ≤ 45	18.9	88.9	17.6	-77	1.08	0.63

LR+: Positive Likelihood Ratio; LR-: Negative Likelihood Ratio; MSRA: Mini Sarcopenia Risk Assessment [5]; NNS: Number Needed to Screen; PPV: Positive Predictive Value; SARC-F: Strength, Assistance with walking, Rise from a chair, Climb stairs and Falls [4]; ^§^ Values are %

Only one person (0.05%) was diagnosed with sarcopenic obesity out of 220 patients with RMDs who were assessed for sarcopenia. Based on the original EWGSOP criteria, the SARC-F presented a sensitivity and a specificity of 22.2% and 75.6%, respectively, while positive (LR+) and negative likelihood values (LR) were 0.9 and 1.03, respectively. The tool’s positive predictive value (PPV) was 15.1%, while the number needed to screen (NNS) was 83.

The 5-item MSRA had 88.9% and 18.9% sensitivity and specificity, respectively, with an LR + of 1.08 and an LR of 0.63. The PPV and NNS were 17.6% and − 77, respectively. Likewise, the 7-item MSRA had sensitivity and specificity values of 91.7% and 9.2%, with LR + and LR of 1.0 and 0.98, respectively. The PPV for the 7-item MSRA was 16.4%, while the NNS was 779.

### Univariate and multivariate logistic regression

The results of the univariate and the multivariate logistic regression analyses are summarized in Table 4. Significant predictors of sarcopenia included having a BMI less than 18 kg/m^2^ (OR: 13.1, 95% CI: 2.78–128) or a BMI greater than 25 kg/m^2^ (OR: 0.05, 95% CI: 0.01–0.16), patient-reported lack of appetite (OR: 3.63, 95% CI: 1.41–8.96), and suffering from RA (OR: 0.31, 95% CI 0.11–0.75), and SSc (OR: 7.66, 95% CI: 2.7–22.1). Other variables, including sex (OR: 1.08, 95% CI: 0.5–2.51), age (OR: 0.99, 95% CI: 0.97–1.01), current corticosteroid use (OR: 1.78, 95% CI: 0.86–3.71), and inflammatory markers such as C-reactive protein (CRP, OR: 0.98, 95% CI: 0.86–1) and erythrocyte sedimentation rate (ESR, OR: 1.0, 95% CI: 0.99–1.02), were not associated with sarcopenia. Similarly, other rheumatic diseases, including SLE, vasculitides, SpA, and PsA, did not demonstrate significant associations with sarcopenia in the present sample. At the multivariate logistic regression, only SSc was associated with an increased risk of sarcopenia (OR: 4.47, 95% CI: 1.44–13.89), while both RA and appetite loss did not reach any significance.

**Table 4:** Univariate and multivariate logistic regression analyses of sarcopenia assessment (Ν = 220)

Variables	Univariate		Multivariate	
	OR (95% CI)	*p* value	OR (95% CI)	*p* value
Sex^§^	1.08 (0.50–2.51)	0.83		
Age (age)	0.99 (0.97–1.01)	0.59		
Underweight^ß^	13.1 (2.78–128)	< 0.001*		
Overweight/obese^ß^	0.05 (0.01–0.16)	< 0.001*		
Smokers^#^	1.33 (0.55–3.00)	0.49		
Dysphagia^#^	1.13 (0.5–2.42)	0.7		
Loss of appetite^#^	3.63 (1.41–8.96)	< 0.001*	2.46 (0.85–6.65)	0.08
CRP (mg/dL)	0.98 (0.86–1.00)	0.65		
ESR (mm/h)	1.00 (0.99–1.02)	0.43		
Corticosteroids use^#^	1.78 (0.86–3.71)	0.11		
RA^#^	0.31 (0.11–0.75)	0.02*	0.42 (0.14–1.04)	0.08
SLE^#^	1.95 (0.71–4.85)	0.17		
Vasculitides^#^	0.97 (0.31–2.55)	0.96		
SpA^#^	0.68 (0.10–2.59)	0.63		
Myositides^#^	1.80 (0.25–8.24)	0.48		
SSc^#^	7.66 (2.70–22.16)	< 0.001*	4.47 (1.44–13.89)	0.01
PsA^#^	0.86 (0.19–2.76)	0.83		
Unidentified rheumatic disease^#^	0.46 (0.02–2.51)	0.47		

CI: confidence intervals; CRP: C-reactive protein; dl: deciliter; ESR: Erythrocyte Sedimentation Rate; h: hour; mg: milligrams; mL: millimeters; OR: odds ratio; PsA: psoriatic arthritis; RA: rheumatoid arthritis; SLE: Systemic Lupus Erythematosus; SpA: spondylarthritis; SSc: Systemic Sclerosis. * *p* < 0.05; ^§^ binary data of men/women; ^ß^ in comparison to normoweight; ^#^ binary data (Yes/No)

## Discussion

To our knowledge, this is the first study evaluating sarcopenia in a sample of patients with broad RMD diagnoses using these specific screening tools. The SARC-F presented higher specificity (75%) than its sensitivity (22%), meaning it consists of a stronger tool for identifying patients without sarcopenia instead of identifying those who have it. In addition, LR + and LR- values and a PPV of 15% demonstrate a high probability that it may misclassify cases. On the other hand, the 5- and 7-item MSRA presented high sensitivity (88% and 91%, respectively) but extremely low specificity (19% and 9%, respectively). This finding indicates that while both tools are effective in detecting individuals with sarcopenia, they can also produce false positive results to a great degree. The LR + and LR- metrics of tests further indicate the poor diagnostic performance of the MSRA in sarcopenia screening. Additionally, the low PPV values (16% for the 7-item and 18% for the 5-item MSRA) emphasize its limited reliability in accurately screening for sarcopenia. The performance of chi-square analyses failed to reveal significant associations between sarcopenia screening and diagnosis. The calculated negative NNS suggests inefficiency in the screening process and may be due to higher sensitivity of the MSRA. Given that all three questionnaires are specifically designed to assess sarcopenia risk, it is reasonable to understand that they are unsuitable for use alone, in patients with RMDs.

Limited research has investigated sarcopenia in RMDs using the SARC-F screening tool, mostly including patients with RA [7, 12, 13], SpA [14], SSc [15] and fibromyalgia [16]. To our knowledge, no studies have utilized the MSRA tool thus far to screen for sarcopenia in this population. Nonetheless, the findings herein align with those in patients with SSc, where the SARC-F alone performed poorly as a screening tool [15] compared with the EWGSOP criteria [3]. This indicates the importance of using the SARC-F as a complementary screening tool, which coincides with its intended purpose when originally developed. In addition, the symptomatology of RMDs involves pain, specifically in the area of the joints [27], which often hinders mobility and limits the ability to perform daily activities [27]. These challenges might influence the SARC-F score as patients are more likely to report difficulty lifting weights, transferring from bed to chair, and climbing stairs [27]. Concerning the MSRA, cultural and dietary factors may affect its performance in Greek inpatients.

Most patients with RMDs suffer from frequent illnesses and infections, increasing the risk of hospitalization. Furthermore, components of the 7-item MSRA, such as dairy and protein consumption questions, may yield greater scores in this population. This is particularly relevant in Greece, where approximately 75% of the population has lactose intolerance [28], and traditional Greek Orthodox lent practices often reduce protein intake [29]. While these factors might result in more patients identified as being at risk, this does not diminish the tool’s value for screening purposes; however, the tool appears to be unsuitable for use alone under such circumstances.

In the present study sarcopenia was positively associated with low/high BMI, specifically with underweight and overweight/obesity. Having a BMI of less than 18 kg/m^2^ presented a 13-fold increase in the odds of presenting sarcopenia. In comparison, a greater BMI denoting overweight (more than 25 kg/m^2^) was associated with lower odds of sarcopenia, as previously reported–though not in the same population [30]–. Moreover, patient-reported loss of appetite was also related to increased odds of sarcopenia. Concerning the underlying disease, RA and SSc demonstrated a notable association with sarcopenia. Individuals with RA have only 31% odds of developing sarcopenia. This result was intriguing because of the underlying inflammation of the RA, as well as joint pain, which usually leads to impaired physical function [31, 32]. More analyses might be needed or consider the treatments. On the other hand, individuals with SSc presented 7.66-fold increased odds of developing sarcopenia. This finding could be due to the gastrointestinal dysmotility and malabsorption patients face, which is exhibited [8] alongside myopenia [33]. Nevertheless, in the present study, inflammation did not contribute to sarcopenia, as shown by CRP and ESR concentrations. Previous research on patients with RA has suggested that high CRP concentrations are related to sarcopenia and low muscle mass [34]. At the same time, in young adults with juvenile idiopathic arthritis, ESR has also been associated with sarcopenia [35]. No other variable was associated with sarcopenia, including sex, age, and corticosteroid use, while the latter presented 78% higher odds of developing sarcopenia but without reaching significance. In the multivariate analysis, SSc emerged as the only significant risk factor, while appetite loss and RA were identified as potential associations, without achieving significance.

Sarcopenic obesity was not prevalent among patients with RMDs in the present sample. Chronic inflammation stimulates the heightened activity of macrophages and lymphocytes, initiating a cascade of reactions that lead to protein breakdown [36]. This accelerates overall protein turnover in the body, contributing to muscle depletion and the progression of cachexia [36]. This protein deficit further elevates metabolic demands, commanding higher energy intake to sustain several physiological functions. If these nutritional needs are not met, patients may experience unvoluntary body weight reduction and muscle loss. This catabolic state might explain why sarcopenic obesity was not observed in this population.

It is well known that RMDs are marked by myopenia [33, 37] and cachexia [38] with low muscle mass being a common underlying factor, ultimately contributing to sarcopenia. Pro-inflammatory cytokines, including interleukin-6 (IL-6) and tumor-necrosis factor-α (TNF-α), are implicated in the pathogenesis of RMDs [39, 40]. Both cytokines are also strongly associated with muscle deterioration and reduced muscle strength [41, 42]. In addition, joint pain–as previously commented – significantly limits mobility, which in turn, accelerates the loss of muscle mass and strength [32]. Consequently, sarcopenia is closely linked to RMDs driven by both physiological mechanisms and clinical manifestations due to the nature of RMDs. Among the treatments used in these conditions, glucocorticoids consist of commonly prescribed medication [43]. This class of drugs induces protein degradation and muscle wasting through a variety of mechanisms [44, 45], adding another factor in the development of sarcopenia in individuals with RMDs. Indeed, research has previously associated the average dose of glucocorticoids in RA with a greater risk of sarcopenia [45]. However, we were unable to analyze the cumulative dose of the participants due to a lack of data. Most of the patients herein exhibited reduced muscle strength as evidenced by low HGS, highlighting the critical need for sarcopenia assessment. Sarcopenia in RMDs contributes to numerous negative outcomes that affect both disease progression and overall well-being. Specifically, it has been linked to malnutrition [9], higher disease activity [7], frailty [11], reduced functional ability [14], poorer disease outcomes [10], and lower bone mineral density, increasing the risk of osteoporosis [14]. Frailty is a major concern in RMDs, with sarcopenia playing a key role, primarily characterized by the loss of skeletal muscle mass and strength [46]. When assessing frailty in this population, and especially in patients with SSc, it is crucial to consider the impact of sarcopenia-related disability [46]. Early identification and timely intervention are essential to mitigate its effects and improve patient outcomes. Currently, sarcopenia screening is not routinely performed in RMD clinics. However, a clear benefit is expected if rheumatologists are educated in screening for sarcopenia and triage patients, as they will be able to support patients better, possibly improve patient functional ability, and reduce the overall economic and disease burden [47].

To protect patients with RMDs against sarcopenia, research has explored nutritional and physical activity interventions. It has been reported that diets enriched in vitamin D and leucine induce improvements in muscle mass and lower limb function in individuals with sarcopenia [48]. Furthermore, a combination of whey protein, vitamin D, and vitamin E supplementation has been shown to enhance muscle mass and improve quality of life [49]. Additionally, β-hydroxy-β-methylbutyrate (HMB) has been highlighted for its potential benefits in addressing frailty among older patients with sarcopenia [50]. With regards to physical activity, it acts in protecting against the development of sarcopenia, increasing both physical performance and muscle strength [51, 52], particularly when paired with dietary supplementation [53]. Further research is required to evaluate the potential interventions for preventing sarcopenia in this population.

### Limitations

Sarcopenia was assessed using anthropometric indices where the FFMI was calculated from body fat assessment using calipers, rather than determined using the gold standard method (dual-energy X-ray absorptiometry). This approach may have over- or under-estimated the prevalence of sarcopenia. To reduce this likelihood, we applied the cut-offs from the EWGSOP 2010 regarding the FFMI. In addition, the validity of both screening tools (SARC-F and MSRA) was investigated based on the EWGSOP criteria [2], which could have impacted the results, making it difficult to reach a robust conclusion. Nevertheless, the findings herein align with those of previous research. In addition, the assessment of sarcopenia could have been based on the EWGSOP criteria of 2019; however, some data were still absent, including the duration and current treatment, which might provide useful insights into physical performance and movement ability. Regarding weight status, there was an imbalance among the three categories, resulting in a wide 95% CI due to challenges encountered during univariate analyses. For this reason, we did not include weight status in the multivariate analysis. Furthermore, the small number of patients in certain disease categories prompted us to analyze the entire sample rather than subdividing it into smaller groups in order to avoid statistical power loss. The small number of patients and the diversity of diseases also acted as a limiting factor in analyzing disease severity and linking it to sarcopenia. Including more patients with certain diseases and increasing the sample size would also enhance the precision of the present analyses. Furthermore, the inclusion of a control group could have generated useful results.

## Conclusions

Sarcopenia is prevalent among patients with RMDs and has a significant impact on their disease outcomes. In this cross-sectional study, both the SARC-F and the MSRA have overestimated the risk of sarcopenia, with the MSRA presenting extremely low sensitivity in detecting individuals with sarcopenia, indicating that it might not be a good screening tool for its assessment within this population. The SARC-F should be used as a complementary tool, as originally designed. A BMI less than 18 kg/m^2^, being diagnosed with SSc, and experiencing loss of appetite appear to be definite contributors to sarcopenia in RMDs. More research is required to clarify the definition of sarcopenia and validate the screening tools for this population.

## Data Availability

The datasets collected for this manuscript are accessible from the corresponding author upon reasonable request.

## Declarations

### Disclaimer

No part of this manuscript, including text and graphics, is copied or published elsewhere in whole or in part.
